# ProtQuant: a tool for the label-free quantification of MudPIT proteomics data

**DOI:** 10.1186/1471-2105-8-S7-S24

**Published:** 2007-11-01

**Authors:** Susan M Bridges, G Bryce Magee, Nan Wang, W Paul Williams, Shane C Burgess, Bindu Nanduri

**Affiliations:** 1 Department of Computer Science and Engineering, Mississippi State University, Starkville, MS, 39762, USA; 2 College of Veterinary Medicine, Mississippi State University, Starkville, MS, 39762, USA; 3 USDA ARS Corn Host Plant Resistance Research Unit, Mississippi State University, Starkville, MS, 39762, USA; 4 Institute for Digital Biology, Mississippi State University, Starkville, MS, 39762, USA; 5 Mississippi Agriculture and Forestry Experiment Station, Mississippi State University, Starkville, MS, 39762, USA

## Abstract

**Background:**

Effective and economical methods for quantitative analysis of high throughput mass spectrometry data are essential to meet the goals of directly identifying, characterizing, and quantifying proteins from a particular cell state. Multidimensional Protein Identification Technology (MudPIT) is a common approach used in protein identification. Two types of methods are used to detect differential protein expression in MudPIT experiments: those involving stable isotope labelling and the so-called label-free methods. Label-free methods are based on the relationship between protein abundance and sampling statistics such as peptide count, spectral count, probabilistic peptide identification scores, and sum of peptide Sequest XCorr scores (ΣXCorr). Although a number of label-free methods for protein quantification have been described in the literature, there are few publicly available tools that implement these methods. We describe ProtQuant, a Java-based tool for label-free protein quantification that uses the previously published ΣXCorr method for quantification and includes an improved method for handling missing data.

**Results:**

*ProtQuant *was designed for ease of use and portability for the bench scientist. It implements the ΣXCorr method for label free protein quantification from MudPIT datasets. *ProtQuant *has a graphical user interface, accepts multiple file formats, is not limited by the size of the input files, and can process any number of replicates and any number of treatments. In addition,*ProtQuant *implements a new method for dealing with missing values for peptide scores used for quantification. The new algorithm, called ΣXCorr*, uses "below threshold" peptide scores to provide meaningful non-zero values for missing data points. We demonstrate that ΣXCorr* produces an average reduction in false positive identifications of differential expression of 25% compared to ΣXCorr.

**Conclusion:**

*ProtQuant *is a tool for protein quantification built for multi-platform use with an intuitive user interface. *ProtQuant *efficiently and uniquely performs label-free quantification of protein datasets produced with Sequest and provides the user with facilities for data management and analysis. Importantly, *ProtQuant *is available as a self-installing executable for the Windows environment used by many bench scientists.

## Background

The expression of the protein complement of the genome, called the proteome, is temporal and cell or tissue-specific. Proteins exist in the cells in physical forms that cannot be predicted from DNA and mRNA analysis[1]. Therefore, direct analysis at the protein level is necessary because proteins are the effectors of function in the cell and are responsible for the phenotype. The goal of proteomics, i.e. study of the proteome, is to directly identify, characterize, and quantify proteins from a particular cell state. We describe the publicly available *ProtQuant *tool for label-free quantification of proteomics datasets.

Multidimensional Protein Identification Technology (MudPIT) coupled with database searching is a common approach in biological studies to identify proteins [2]. MudPIT involves site-specific proteolytic digestion of proteins to peptides, separation of peptides by two-dimensional liquid chromatography (LC) (strong cation exchange and reverse phase), and analysis of peptides by tandem mass spectrometry (MS/MS), followed by database searching for protein identification. The databases used for matching MS/MS spectra are *in silico *digested with the same site-specific protease and include all possible "fingerprints" for peptides for all proteins in the database. Protein identification using database searching algorithms like Sequest [3], MASCOT [4] and OMSSA [5] are based on thresholds for specific scoring parameters for each algorithm that are used to filter the peptide identifications most likely to be correct. For example, the Sequest cross correlation coefficient score (XCorr) and delta Cn (ΔC_n_) represent sensitivity and specificity respectively for peptide identification and thresholds for these two scores are used for filtering out false positives [6]. Identification of protein specific peptides confirms the presence of the protein in the sample. It is important to note that only 10–50% of spectra assignments generated in LC-MS/MS experiments are actually correct [7] and a majority of peptide assignments to spectra are removed by filtering.

For meaningful modelling of biological data, mere identification of proteins from a sample is not sufficient; quantitative analysis is required. Non-gel based quantitative proteomics methods can be broadly categorized into isotopic and isotope-free methods. Isotopic methods like ICAT [8], iTRAQ [9] and ^18^O [10] involve labelling peptides from different experimental conditions with different stable isotopes and introducing predictable mass differences between identical peptides. The ratios of the ion intensities for labelled pairs of peptides are used to quantify the relative abundance of the proteins.

Isotope-free methods use the observed parameters for protein identification as well as sample replication to measure changes in relative protein abundance. Examples include: the peptide count [11], spectral count [12], sequence coverage [13], and exponentially modified protein abundance index [14]. We have previously shown that the Sequest cross correlation (XCorr) is inherently quantitative and can be used for non-isotopic quantitative MuDPIT proteomics [15] and that comparison of the sum of XCorr values associated with peptides used to identify a protein (ΣXCorr) in treatment and control can be used for relative protein quantification.

Here we describe a new tool, *ProtQuant*, for ΣXCorr quantification of label-free 2D LC MS/MS data analyzed by TurboSEQUEST™ (Bioworks Browser 3.2 ThermoElectron). *ProtQuant *is a stand alone, platform independent Java program and is available as a self-installing executable for Windows platforms. It has a graphical user interface and can handle multiple data input formats and multiple replicates of biological experiments.

In addition, ProtQuant implements an improved method imputing missing data values when computing the ΣXCorr – we call the improved method ΣXCorr*. Because MudPIT mass spectrometry is based on sampling from a complex protein mixture, the peptides that are identified from one replicate to another are extremely variable. Durr et al. [16] have reported that only ~66% of the peptides identified in any one replicate are present in a second replicate and that up to ten replicates are required to ensure that no new peptides are identified. However, due to the time and expense involved, researchers rarely collect more than three replicates and smaller numbers are common. None of the isotopic or non-isotopic methods address the issue of "missing" mass spectra. Missing mass spectra occur due to the inherent limitations of mass spectrometers, the probabilistic nature of sampling, and the fact that the cut-offs used to determine "true" assignments of peptides to mass spectra are not truly biological. Such data gaps are ignored or replaced by zeros in the differential analyses of non-isotopic proteomics. Both approaches can bias the comparisons and increase the number of significantly differentially-expressed proteins identified by statistical tests. The ΣXCorr* method uses "below threshold" peptide XCorr scores to impute "missing values" and reduce false positive identifications of differential expression.

## Results

**Figure 1:**
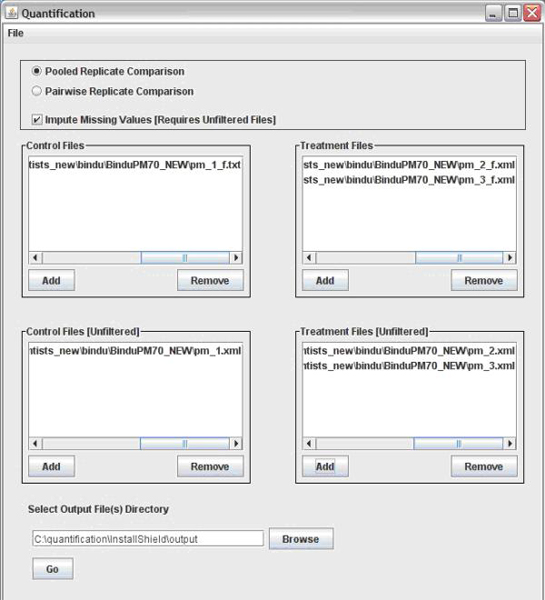
ProtQuant user interface.

*ProtQuant *provides proteomics researchers with a convenient tool for protein quantification of a 2D LC ESI MS/MS experiment and it runs on the Windows platform used by many bench scientists. *ProtQuant *has a graphical user interface, accepts multiple file formats, and is not limited by the number of replicates, the number of treatments, or size of the input files. The Sequest XML format must be used when the size of the dataset exceeds the maximum size of an Excel spreadsheet. We provide a program that can generate tab-delimited text files from the large XML files and that scientists can view and edit using a text editor such as Notepad. The *ProtQuant *graphical user interface is illustrated in Figure 1. The user selects the file input format, the Sequest generated files to be compared, and a confidence level for ANOVA analysis. Files representing multiple conditions, as well as multiple replicates for each condition, can be processed. One condition is designated as the control and the remaining conditions as treatments. The user can select the ΣXCorr (without imputation of missing values) or ΣXCorr* quantification methods. Replicates can be processed as pairs from the control and treatment or replicates from each treatment can be pooled (the default).

**Figure 2:**
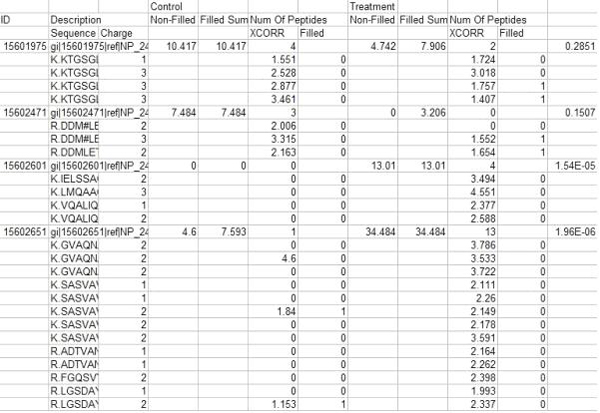
Sample ProtQuant output.

*ProtQuant *loads the input files selected using a browse facility, matches the proteins found in each file, and constructs a master peptide list for each protein. Missing values are imputed if requested, and the ΣXCorr for each protein in the control and each treatment is computed. *ProtQuant *also computes the statistical significance of differential expression of control and treatment for each protein using one-way ANOVA. The program generates a tab delimited text file as output that can easily be imported into Excel for further analysis by the scientist (see Figure 2). Statistical significance levels are computed only for those proteins where at least three XCorr values were used to compute the ΣXCorr.

In developing ProtQuant, we address the issue of missing mass spectral identifications when comparing two proteomic datasets to determine significant changes in relative protein expression. To understand the rationale for designing *ProtQuant *it is first necessary to understand tandem mass spectrometry-based protein identifications and their limitations. When processing cDNA micro-arrays and other large scale quantitative mRNA analyses that rely on nucleotide sequence identity and hybridization, missing values originate from random imperfections at the level of chip production, treatment, hybridization, and scanning [17,18]. Tandem mass spectrometry-based identifications, on the other hand, are inferred based on the quality of matches between observed spectra and predicted sequence-specific patterns and the concept of "missing values" is fundamentally different. The two most commonly used algorithms for doing tandem mass spectrometry-based identifications are Mascot [4] and Sequest [19]. Both rely on the principle of first using the specific mass (± the known mass error) of the precursor ion (i.e. before fragmentation) to generate a short-list of all peptide sequences in the database with masses within the range that could be derived from that precursor ion. Then the algorithms score the correlations of the associated tandem mass-spectra (generated from precursor ion fragmentation) for each peptide in the short list. The scores include:

1. A correlation score based on the frequency and intensity of the ("y", "b" and sometimes other) ions in the tandem mass spectra ("XCorr" for Sequest and "Ion Score" for Mascot) and

2. A relative score based on the rank of the correlation scores for each match on the short-list ("ΔCn" for Sequest and "homology factor" for Mascot).

Then identifications are decided based on "cut-off" scores determined either by applying scores based on how the scores performed on an unrelated training data set [19], or a probability based determination based on searching against a "decoy data-base" [20]. Because of the great variability in the peptides detected by mass spectrometry even among technical replicates [16], a comparison of peptide identifications for a specific protein in a control and treatment often exhibit substantial differences. When using peptide statistics for relative quantification, it is important to determine, to the extent possible, if the peptides found in one sample but not the other are truly missing or if they were present but were below the cut-off threshold for identification. *ProtQuant *makes use of the quantitative data provided from mass spectra below the identification threshold that current non-isotopic quantification methods are missing. Note that methods based on spectral counts or peptide counts cannot use this information since these methods rely on counting and not a quantitative peptide score. For a peptide that occurred in the treatment or control but not the other, we ask the following question: Was a tandem mass spectrum present that correlates with the peptide but with an XCorr value below the user defined threshold for identification? If so, we use this XCorr value to impute a score for the peptide. Conversely, if there is no such mass spectrum, then we replace the missing value with zero.

**Figure 3:**
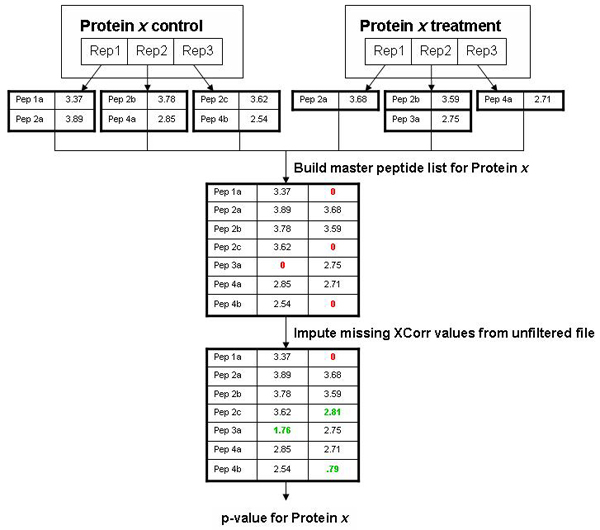
**ProtQuant method for handling missing data**. Red numbers indicate missing values. Values from the unfiltered file used to replace missing values are in green.

Figure 3 illustrates this process in more detail. For each experiment, we are interested in comparing a control and treatment where both datasets have replicates. We generate two Sequest output files for each replicate – one that is generated with user-defined thresholds for XCorr values to select peptides used for protein identification (called the filtered data) and one that is generated with no thresholds (called unfiltered data). Note that the number of peptide assignments to spectra that are "filtered out" is very large and in most datasets will substantially exceed the number of assignments that have scores sufficiently high to be used for protein identification. The unfiltered data is used only for imputing missing values and not for peptide identification and the below threshold values that are used to impute missing values constitute a very small percentage of the unfiltered matches (<0.1%).

For each protein, based on the accession number, all peptides used to identify that protein (those present in the filtered data) in all replicates under consideration are combined into a master peptide list for that protein. If a peptide is present in the filtered data in either the control or treatment (but not both), we search to see if that peptide is present in the unfiltered data. If it is, we use the largest below-threshold XCorr value for that peptide from the set in which it did not score above the threshold. Such XCorr values occur when the nominated peptide was present but the quantity in the sample was too low to generate ion frequency and intensity to score above the cut-off threshold. If the peptide is not represented by a tandem mass spectra in the unfiltered data, a value of zero is used. Using the imputed values provides a smooth transition for XCorr values between the threshold and zero and provides datasets that can be tested with parametric statistics. We use one-way analysis of variance, a published statistical method for using ΣXcorr for relative quantification of protein expression.

We compared the performance of the ΣXCorr and ΣXCorr* methods for label-free protein quantification with 2D LC ESI MS/MS data using *Pasteurella multocida *cell lysate sample that was spiked with five different concentrations (3, 6, 12, 120, and 1200 pmol) of BSA, lysozyme, and cytochrome C as described in [15]. Each dilution represents a technical replicate of the *P. multocida *sample. Regression analysis of both ΣXCorr and ΣXCorr* as a function of protein concentration yielded virtually identical R^2 ^values of over 98% as described in [15]. We conclude that imputation of missing values using below threshold scores does not negatively impact the ability to detect differential expression of proteins.

**Figure 4:**
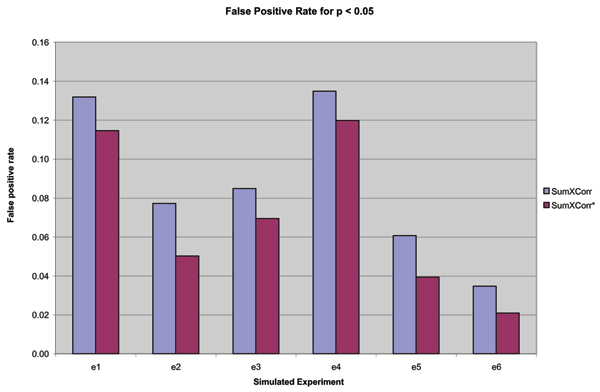
**False positive identification rates for simulated experiments**. For each simulated experiment, two of the spiked samples were chosen as the control and two others as the treatment (see Table 1 for the pairings). All identifications of differential expression at p ≤ 0.05 represent false positives.

We also used the spiked dataset to evaluate the false positive identification rate of the two methods. Because the amount of protein spiked into each of the technical replicates was very small and has a negligible effect on the overall protein concentrations, the concentration of non-spiked proteins should be the same in all replicates. The spectra from each of the spiked samples was searched against the *Pastuerella multocida *protein databases using Sequest as described in [15]. We constructed simulated experimental datasets by labelling two of the spiked samples as "control" and two others as "treatment". Six different combinations were used to construct 6 simulated experimental datasets. The concentration of all proteins in the controls and treatments should be the same in the simulated experiments and the variation in the peptides and their XCorr values that occurred between the simulated controls and treatments was due to random fluctuations. Thus any proteins that were identified as being differentially expressed at a specified statistical level are considered false positive identifications of differential expression. Table 1 shows the pairings that we used to construct each of the 6 sets and Figure 4 gives the resulting false positive identification rates for both ΣXCorr and ΣXCorr* for p ≤ 0.05. Although the false positive identification rate varies substantially from one simulated experiment to another, the rate is always reduced using ΣXCorr* with an average reduction of 25.5%.

**Table 1:** Simulated experiments used to evaluate false positive identification rates of ΣXCorr and ΣXCorr*.

Simulated Experiment	Spiked Samples Used as Replicates for Control	Spiked Samples Used as Replicates for Treatment
1	1200 & 3	120 & 12
2	1200 & 3	120 & 6
3	1200 & 6	120 & 3
4	1200 & 6	12 & 3
5	1200 & 12	120 & 6
6	1200 & 12	120 & 3

Five technical replicates of a P. multocida sample were spiked with different concentrations (3, 6, 12, 120, & 1200 pmol) of standards. Pairs of the spiked samples were used to represent "control" and "treatment" in simulated experiments. Any non-spiked proteins that are identified as differentially expressed are false positives (see Figure 4).

The bacterial proteomics data set, *Streptococcus pneumoniae *was grown in triplicate, either in the presence or absence of iron in the growth medium. Total proteins were isolated and proteomic analysis was carried out as previously described [15]. Tandem mass spectra were searched against all proteins from *S. pneumoniae *Tigr4. Of the 171 proteins identified, *ProtQuant *without imputation of missing values found 21% to be differentially expressed while ProtQuant with imputation of missing values found only 8% to be significantly different between iron rich and iron restricted growth conditions. The proteins identified as differentially expressed with ΣXCorr* were a subset of those identified as differentially expressed by ΣXCorr, i.e. no new false positives are introduced with imputation of missing values. We conclude that ΣXCorr* significantly reduces false positive identifications without introducing false negative identifications of differential expression.

## Discussion

The availability of high throughput methods such as 2D LC ESI MS/MS has made it possible to characterize changes in the proteome of an organism, tissue, or cell under specified conditions. In addition to determining the identity of proteins present under different conditions, it is also essential to be able to quantify changes in protein expression. Label-free methods may be preferred over labelling methods because they are faster, less expensive, generally provide greater proteome coverage and do not exhibit problems found with incomplete labelling. *ProtQuant *is a reliable computational tool for relative protein quantification from isotope-free MuDPIT data analyzed by Sequest. Unlike methods for label-free quantification such as spectral count or peptide count that depend on statistics of counts, the ΣXCorr method uses a quantitative score that is associated with each peptide identification for protein quantification. ΣXCorr* also makes use of below threshold scores that cannot be used for identification, but that provide useful information for quantification.

Unlike gene expression data where missing values are due to technical failures, low signal-to-noise ratio, and measurement error, missing values in MudPIT data are due to the probabilistic nature of peptide observation by the mass spectrometer. Methods such as the kNNimpute procedure for imputing missing values that are used in SAM and many other gene expression analysis software packages [17,18] do not address the same problem. These methods estimate missing values for genes based on the values available for genes that behave in a similar manner (k-nearest neighbors based on Euclidean distance of expression vectors for the genes excluding the missing value). However, in the case of proteomics experiments, the problem is quite different because the peptide identifications are based on matching real spectra to theoretical spectra generated by *in silico *generated peptides. Peptides may be truly missing – that is they may not be present in a sufficient quantity to be detectable. In these cases, it is correct to use 0 as the score for the "missing peptide". However, the peptide may be present but may not have generated a signal of sufficient strength to score above the threshold. In these cases, the below-threshold score provides information about relative peptide quantity and can therefore be used for protein quantification.

Future planned extensions to ProtQuant include addition of options for other label-free quantification methods such as spectral counting, and additional methods for statistical analysis such as Monte Carlo statistics. In addition, we are developing a new method for peptide validation that will produce output in a form that can be used by ProtQuant tool for relative quantification. Integration of ProtQuant with other tools that perform clustering, and GO annotation of proteins, pathway analysis is also planned.

## Conclusion

*ProtQuant *has a user-friendly interface and robust capabilities for managing files. *ProtQuant *uniquely performs label-free relative quantification and is available for the Windows platform used by many researchers.

## Methods

### Implementation of *ProtQuant*

*ProtQuant *is implemented in Java 5 for platform independence. A self-installing executable for Windows has been generated using Macrovision InstallShield. Instructions for installing and using the tool in a Linux environment are also available. ANOVA analysis is done using a library from the R statistical package . Because of the size of the datasets that *ProtQuant *must handle, MySQL is used for data storage and efficient data manipulation. *ProtQuant *uses the file extension of input files to determine the format. *ProtQuant *includes a custom built parser for XML files.

### Protein identification

2D LC ESI MS/MS data was analyzed as published by Nanduri et al [15] to test the system. Regression analysis of both ΣXCorr and ΣXCorr* with protein concentration and the analysis of false positive identifications for the two methods was conducted with this dataset. For the bacterial dataset, database searches were conducted against all proteins from *S. pneumoniae *TIGR4 using TurboSEQUEST™ (Bioworks Browser 3.2; ThermoElectron). Trypsin digestion was applied *in silico *including differential modifications of cysteine (carboxyamidomethylation) and methionine (oxidation) in the search criteria. Peptides were deemed to have identified a protein from the database when they are at least 6 amino acids long, with a XCorr of 1.5, 2.0, and 2.5 for +1,+2, and +3 charged ions, respectively, and a delta Cn value of 0.1 or greater [21].

## Competing interests

The authors declare that they have no competing interests.

## Authors' contributions

SMB, GBM designed and implemented ProtQuant. NW developed an early prototype of the tool. BN and SCB developed the method of using the unfiltered files to find missing values and contributed the data used for development and testing. WPW supported development of the tool. BN conducted extensive testing of the tool. SMB, GBM, WPW, BN and SCB contributed to writing the manuscript.

## References

[B1] Ong SE, Mann M (2005). Mass spectrometry-based proteomics turns quantitative. Nat Chem Biol.

[B2] Washburn MP, Wolters D, Yates JR (2001). Large-scale analysis of the yeast proteome by multidimensional protein identification technology. Nat Biotechnol.

[B3] Eng JK, McCormack AL, Yates JR (1994). An approach to correlate tandem mass spectral data of peptides with amino acid sequences in a protein database. J Am Soc Mass Spectrom.

[B4] Perkins DN, Pappin DJ, Creasy DM, Cottrell JS (1999). Probability-based protein identification by searching sequence databases using mass spectrometry data. Electrophoresis.

[B5] Geer LY, Markey SP, Kowalak JA, Wagner L, Xu M, Maynard DM, Yang X, Shi W, Bryant SH (2004). Open mass spectrometry search algorithm. J Proteome Res.

[B6] Keller A, Nesvizhskii AI, Kolker E, Aebersold R (2002). Empirical statistical model to estimate the accuracy of peptide identifications made by MS/MS and database search. Anal Chem.

[B7] Elias JE, Haas W, Faherty BK, Gygi SP (2005). Comparative evaluation of mass spectrometry platforms used in large-scale proteomics investigations. Nat Methods.

[B8] Gygi SP, Rist B, Gerber SA, Turecek F, Gelb MH, Aebersold R (1999). Quantitative analysis of complex protein mixtures using isotope-coded affinity tags. Nat Biotechnol.

[B9] Ross PL, Huang YN, Marchese JN, Williamson B, Parker K, Hattan S, Khainovski N, Pillai S, Dey S, Daniels S (2004). Multiplexed protein quantitation in Saccharomyces cerevisiae using amine-reactive isobaric tagging reagents. Mol Cell Proteomics.

[B10] Stewart II, Thomson T, Figeys D (2001). 18O labeling: a tool for proteomics. Rapid Commun Mass Spectrom.

[B11] Gao J, Opiteck GJ, Friedrichs MS, Dongre AR, Hefta SA (2003). Changes in the protein expression of yeast as a function of carbon source. J Proteome Res.

[B12] Liu H, Sadygov RG, Yates JR (2004). A model for random sampling and estimation of relative protein abundance in shotgun proteomics. Anal Chem.

[B13] Florens L, Washburn MP, Raine JD, Anthony RM, Grainger M, Haynes JD, Moch JK, Muster N, Sacci JB, Tabb DL (2002). A proteomic view of the Plasmodium falciparum life cycle. Nature.

[B14] Ishihama Y, Oda Y, Tabata T, Sato T, Nagasu T, Rappsilber J, Mann M (2005). Exponentially modified protein abundance index (emPAI) for estimation of absolute protein amount in proteomics by the number of sequenced peptides per protein. Mol Cell Proteomics.

[B15] Nanduri B, Lawrence ML, Vanguri S, Burgess SC (2005). Proteomic analysis using an unfinished bacterial genome: the effects of subminimum inhibitory concentrations of antibiotics on Mannheimia haemolytica virulence factor expression. Proteomics.

[B16] Durr E, Yu J, Krasinska KM, Carver LA, Yates JR, Testa JE, Oh P, Schnitzer JE (2004). Direct proteomic mapping of the lung microvascular endothelial cell surface in vivo and in cell culture. Nat Biotechnol.

[B17] Hua D, Lai Y (2007). An ensemble approach to microarray data-based gene prioritization after missing value imputation. Bioinformatics.

[B18] Scheel I, Aldrin M, Glad IK, Sorum R, Lyng H, Frigessi A (2005). The influence of missing value imputation on detection of differentially expressed genes from microarray data. Bioinformatics.

[B19] Eng JK, McCormack AL, Yates JRI (1994). An approach to correlate tandem mass spectral data of peptides with amino acid sequences in a protein database. Journal of the American Society of Mass Spectrometry.

[B20] Elias JE, Gygi SP (2007). Target-decoy search strategy for increased confidence in large-scale protein identifications by mass spectrometry. Nature methods.

[B21] Nanduri B, Lawrence ML, Boyle CR, Ramkumar M, Burgess SC (2006). Effects of subminimum inhibitory concentrations of antibiotics on the Pasteurella multocida proteome. J Proteome Res.

